# 327. Use of multiple pharmacies by people living with HIV: a qualitative analysis

**DOI:** 10.1093/ofid/ofad500.398

**Published:** 2023-11-27

**Authors:** Eric G Sahloff, Joan Duggan

**Affiliations:** Univ of Toledo, Toledo, OH; University of Toledo Medical Center, Toledo, Ohio

## Abstract

**Background:**

Multiple pharmacy use has been linked to decreased patient adherence, inappropriate drug use, adverse drug reactions, and increased mortality. Despite negative outcomes, multi-pharmacy use has increased and nearly half of all patients in the US now use multiple pharmacies. In this study, the attitudes towards multiple pharmacy use (MPU) versus single pharmacy use (SPU) for PLWH (people living with HIV) were assessed.

**Methods:**

A convenience sample of patients in a single midwestern Ryan White clinic was surveyed. There were 24 questions with two additional questions for users of multiple pharmacies. Questions were related to patient demographics, current CD4 count (CD4), viral load (VL) status, amount of time to receive medications, missed doses, comorbidities, and patient preferences. A 5-point Likert Scale (LS; 1 = strongly disagree, 5 = strongly agree) was used to assess reasons for SPU versus MPU, including for MPU if antiretroviral medications were filled separately.

**Results:**

142 patients participated (69 SPUs; 73 MPUs). Few differences between SPUs and MPUs for demographic indicators were identified (Table 1). SPUs were more likely to use private/commercial insurance versus government-sponsored insurance and use fewer non-HIV medications. There was no difference between groups in self-reported undetectable VL. There was a trend towards increased recall of self-reported CD4 and VL status for SPUs vs MPUs. SPUs indicated that proximity (LS weighted average (WA) = 3.64), getting all meds in one place (LS WA = 4.45), and the identification of adverse effects (LS WA = 4.17) and drug-drug interactions (LS WA = 4.06) as reasons for preferring to use a single pharmacy. For MPUs who indicated they used a separate pharmacy specifically for antiretroviral medications, the need to keep HIV status private (LS weighted average = 3.65) was noted as a significant reason.

**Table 1.**
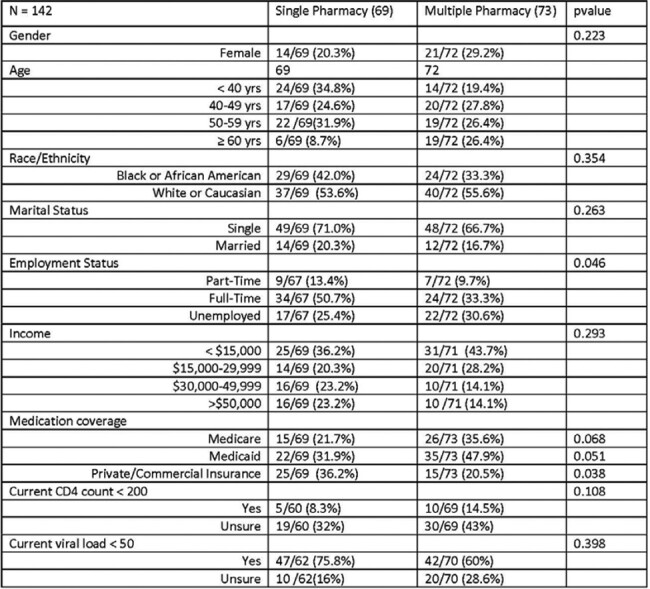
Respondents Demographics

**Table 1:**
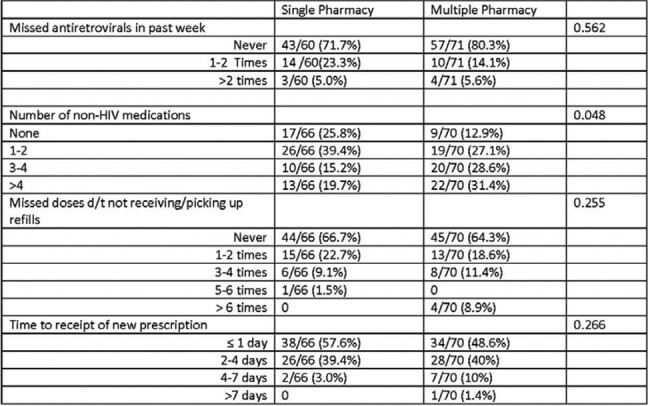
(cont). Respondent Demographics

**Conclusion:**

SPUs chose a single pharmacy for reasons of convenience and safety. MPUs who chose to fill their antiretroviral therapy at a separate pharmacy stated concerns for stigma/privacy as a major contributing factor. Patient preferences should be strongly considered regarding SPU or MPU. Interestingly, MPUs were less likely to be aware of their clinical status.

**Disclosures:**

**All Authors**: No reported disclosures

